# Non-alcoholic fatty liver disease and the development of nephrolithiasis: A cohort study

**DOI:** 10.1371/journal.pone.0184506

**Published:** 2017-10-26

**Authors:** Seolhye Kim, Yoosoo Chang, Eunju Sung, Cheol Hwan Kim, Kyung Eun Yun, Hyun-Suk Jung, Hocheol Shin, Seungho Ryu

**Affiliations:** 1 Center for Cohort Studies, Total Healthcare Center, Kangbuk Samsung Hospital, Sungkyunkwan University School of Medicine, Seoul, South Korea; 2 Department of Occupational and Environmental Medicine, Kangbuk Samsung Hospital, Sungkyunkwan University School of Medicine, Seoul, South Korea; 3 Department of Clinical Research Design & Evaluation, SAIHST, Sungkyunkwan University, Seoul, South Korea; 4 Department of Family Medicine, Kangbuk Samsung Hospital, Sungkyunkwan University School of Medicine, Seoul, South Korea; Università degli Studi di Palermo, ITALY

## Abstract

**Background:**

Non-alcoholic fatty liver disease (NAFLD), a hepatic manifestation or precursor of metabolic syndrome, may increase nephrolithiasis, a renal manifestation of insulin resistance, but the prospective association between NAFLD and incident nephrolithiasis has not been evaluated. We examined the association of NAFLD with the development of nephrolithiasis in a large cohort of Korean men and women.

**Methods:**

We performed a cohort study of 208,578 Korean adults who underwent a health checkup examination between January 2002 and December 2014 and were followed-up annually or biennially through December 2014. NAFLD was defined as the presence of fatty liver in the absence of excessive alcohol use or other identifiable causes. Fatty liver and nephrolithiasis were determined based on ultrasonographic findings. We used a parametric Cox model to estimate the adjusted hazard ratios (HRs) of nephrolithiasis according to the presence of NAFLD.

**Results:**

During 1,054,887.6 person-year of follow-up, 16,442 participants developed nephrolithiasis. After adjusting for age, center, year of screening exam, smoking status, alcohol intake, physical activity, education level, body mass index, history of hypertension and diabetes, HOMA-IR, uric acid and C-reactive protein, male participants with NAFLD had a significantly increased risk of nephrolithiasis than those without NAFLD (adjusted HR 1.17, 95% CI 1.06–1.30). However, no association between NAFLD and nephrolithiasis was observed in women (adjusted HR 0.97, 95% CI 0.81–1.16).

**Conclusions:**

In this large cohort study of young and middle-aged Koreans, NAFLD was significantly associated with an increased incidence of nephrolithiasis in men but not in women.

## Introduction

Nonalcoholic fatty liver disease (NAFLD) is the most common cause of chronic liver disease worldwide [1]. NAFLD is diagnosed by evidence of steatosis by imaging or histology without secondary causes [2]. It exhibits a wide spectrum of liver disease, ranging from simple steatosis to cirrhosis and hepatocellular carcinoma. Aside from its potential to progress to cirrhosis, liver failure, or hepatocellular carcinoma, which ultimately require liver transplantation, NAFLD is also related to extrahepatic diseases, such as cardiovascular disease, type 2 diabetes, obesity, dyslipidemia, and kidney dysfunction [3, 4].

Nephrolithiasis is a common problem with significant health and economic burden and its prevalence and incidence is increasing globally [5]. The occurrence of nephrolithiasis is costly due to both medical treatment and time lost from work [6, 7]. Recently, nephrolithiasis is considered a systemic disorder associated with chronic kidney disease, bone disease, coronary artery disease, hypertension, and type 2 diabetes mellitus [8, 9]. Moreover, studies have suggested that metabolic syndrome is associated with the occurrence of nephrolithiasis [10]. NAFLD is closely associated with insulin resistance and MetS [11], and has even been considered a precursor of MetS [12]. We hypothesized that NAFLD contributes to the formation of nephrolithiasis. Until now, the association between NAFLD and nephrolithiasis has remained largely unexplored. A cross-sectional study by Einollahi *et al*. reported a positive association between NAFLD and nephrolithiasis [13] without controlling for possible confounders, limiting its ability to establish a temporal and independent association between NAFLD and nephrolithiasis. To date, no cohort study has evaluated the effect of NAFLD on the development of nephrolithiasis.

Therefore, we examined the association of NAFLD with the development of nephrolithiasis in a large cohort of Korean men and women free of nephrolithiasis at baseline who participated in a health screening examination program.

## Materials and methods

### Study population

This study was part of The Kangbuk Samsung Health Study. The Kangbuk Samsung Health Study was a cohort study of Korean men and women aged 18 years or over who completed a comprehensive annual or biennial examination at Kangbuk Samsung Hospital Total Healthcare Centers in Seoul and Suwon, South Korea [14, 15]. Employees of various companies and local governmental organizations and their spouses composed over 80% of this large cohort. The Industrial Safety and Health Law in South Korea requires annual or biennial free health check-ups of all employees. The rest of participants voluntarily underwent health check-up.

The present analysis includes all study participants who completed a comprehensive examination from January 2002 to December 2014 and who had at least one follow-up visit through December 31, 2014 (*n* = 263,243).

**Fig 1:**
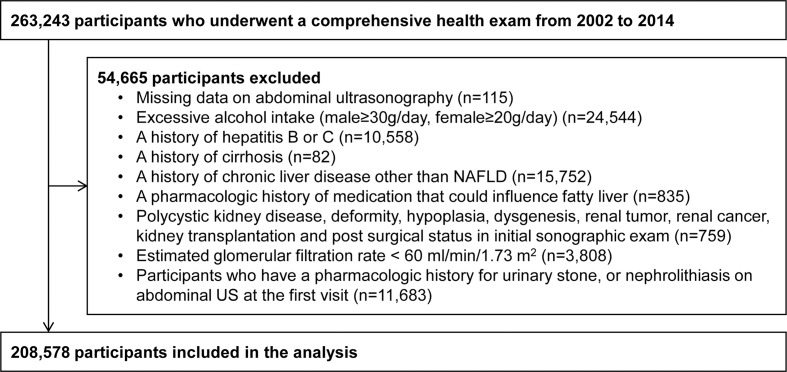
Flow-chart of the included participants.

Among those participants, 54,665 were excluded as follows: 115 had missing abdominal ultrasonography (US) data; 24,544 participants had excessive alcohol intake (male≥30g/day, female≥20g/day); 10,558 participants had hepatitis B or C; 82 participants had cirrhosis; 15,752 participants had chronic liver disease other than NAFLD; 835 participants had a pharmacologic history of medication that could influence fatty liver; 759 participants had polycystic kidney disease, deformity, hypoplasia, dysgenesis, renal tumor, renal cancer, kidney transplantation and post surgical status in initial sonographic exam; 3,808 participants had a glomerular filtration rate (GFR) < 60 ml/min/1.73m2; and 11,683 participants already had a pharmacologic history for urinary stone, or nephrolithiasis on abdominal US at the first visit. Because some participants met more than one exclusion criteria, 208,578 participants were included in this study. (Fig 1) The study was approved by the Institutional Review Board of Kangbuk Samsung Hospital, which waived the requirement for informed consent due to the use of de-identified data.

### Measurement

All examinations were completed at Kangbuk Samsung Hospital Health Screening Center clinics in Seoul and Suwon. Data on demographic characteristics, smoking status, alcohol consumption, physical activity, educational level, medication use, and medical history of hypertension, diabetes, liver disease, and kidney stones were collected by standardized, self-administered questionnaires as previously described [14, 15]. Smoking status was categorized as never, former, and current smokers. Alcohol consumption was categorized as ≤10 grams/day and >10 grams/day. The weekly frequency of moderate- or vigorous-intensity physical activity were also assessed and categorized as < 3 or ≥ 3 times per week.

Height, weight, and sitting blood pressure (BP) were measured by trained nurses. Height was measured to the nearest 1 cm using a stadiometer with the participant standing with naked feet. Weight was measured to the nearest 0.1 kg on a bioimpedance analyzer (InBody 3.0 and Inbody 720, Biospace Co., Seoul Korea) validated for reproducibility and accuracy of body composition measurements [16] and calibrated every morning before examination. Body mass index (BMI) was calculated as weight in kilograms divided by height in meters squared and was classified according to Asian-specific criteria, as follows [17]: underweight, BMI <18.5 kg/m^2^; normal weight, BMI of 18.5 to 23 kg/m^2^; overweight, BMI of 23 to 25 kg/m^2^; and obese, BMI ≥25 kg/m^2^.

After a minimum of 10 hours of fasting, blood specimens were taken from the antecubital vein. Blood tests included total cholesterol, low density lipoprotein-cholesterol (LDL-C), high density lipoprotein-cholesterol (HDL-C), triglyceride, aspartate aminotransferase (AST), alanine transaminase (ALT), gamma-glutamyltransferase (GGT), fasting glucose, uric acid, high sensitive C-reactive protein (hsCRP), and creatinine. The estimated GFR was calculated by using the Modification of Diet in Renal Disease formula [18]. Homeostasis model assessment of insulin resistance (HOMA-IR), an index used to quantify beta-cell function and insulin resistance, was calculated by multiplying fasting insulin (mg/dL) by fasting glucose (mg/dL), then dividing by a correction factor of 405 [19].

Abdominal US was undertaken by experienced radiologists who were unaware of the aim of the study using a Logic Q700 MR 3.5 MHz transducer (GE, Milwaukee, WI, USA). All abdominal US were performed in a standard fashion with the participant in the supine position and the right arm raised above the head. NAFLD was diagnosed according to the following criteria: (1) the presence of a diffuse increase in fine echoes in the liver parenchyma compared with the kidney or spleen, (2) no excessive alcohol consumption (a threshold of 20g/day for women and 30g/day for men), (3) and the absence of competing causes of hepatic steatosis such as viral hepatitis or a pharmacologic history that could influence fatty liver [20, 21]. When hyperechoic structures causing acoustic shadowing were seen in the collecting system, we diagnosed it as nephrolithiasis [22, 23].

### Statistical analyses

All data were presented separately by sex as mean (standard deviation), median (interquartile range), or percentage. Characteristics of the study participants were explored according to the presence of NAFLD. Continuous variables were compared by using a *t*-test and discrete variables were compared by using a chi-square test.

The primary endpoint of this study was the development of nephrolithiasis on sonographic exam. Each subject was followed from the baseline exam either until the development of nephrolithiasis or the last health exam conducted prior to December 31, 2014, whichever came first. Incidence density was calculated as the number of cases divided by person-years during the follow-up period. Because we knew that nephrolithiasis occurred between two visits but not the exact time of occurrence, we used a parametric Cox model to account for the interval censoring (*stpm* command in Stata) [24]. In these models, the baseline hazard function was parameterized with restricted cubic splines in log time with four degrees of freedom.

We calculated hazard ratio(HR) and 95% confidence intervals (CI) for incident nephrolithiasis. The models were initially adjusted for age, and then further adjusted for center, year of screening exam, smoking status, alcohol intake, physical activity, education level, BMI, and history of hypertension and diabetes (Model 1). Finally, the model 2 was further adjusted for HOMA-IR, uric acid, and hsCRP. We assessed the proportional hazards assumption by examining graphs of estimated log (-log) survival. To determine linear trends of incidence, the number of categories was used as a continuous variable and tested on each model.

To explore whether the association between NAFLD and nephrolithiasis differs, we performed stratified analyses in pre-specified subgroups defined by age (<50 vs. ≥50 years), alcohol intake (<10 vs. ≥10g/day), smoking status (never vs. current smokers), HOMA-IR (<2.5 vs. ≥2.5), BMI (<25 vs. ≥25kg/m^2^), and hsCRP (<1.0 vs. ≥1.0mg/l). Interactions between NAFLD and subgroup characteristics were tested using likelihood ratio tests comparing models with and without multiplicative interaction terms.

Statistical analyses were carried out using STATA version 14.0 (StataCorp LP, College Station, TX, USA), All *p*-values of less than 0.05 were considered statistically significant.

## Results

Among the 208,578 participants, there were 112,324 men (53.9%) and 96,254 women (46.1%). Table 1 shows the baseline characteristics of the study participants. The prevalence of NAFLD in males was higher than that in females. NAFLD was positively associated with age, BMI, glucose, uric acid, total cholesterol, LDL-C, triglyceride, AST, ALT, GGT, HOMA-IR, and hsCRP, whereas it was negatively associated with HDL-C and vigorous exercise.

**Table 1:** Baseline characteristics of study participants by non-alcoholic fatty liver disease and sex.

Characteristics	Men		p	Women		p
	No NAFLD	NAFLD		No NAFLD	NAFLD	
**Number**	70,954	41,370		86,660	9,594	
**Age (years)**^**a**^	36.8 (7.6)	37.9 (7.6)	<0.001	36.7 (7.4)	42.8 (10.2)	<0.001
**BMI (kg/m**^**2**^**)**	23.2 (2.4)	26.1 (2.7)	<0.001	21.3 (2.5)	25.6 (3.3)	<0.001
**Obesity (%)**	21.8	63.4	<0.001	8.1	52.3	<0.001
**Current smoker (%)**	42.1	42.2	<0.001	3.9	3.6	0.255
**Alcohol intake (%)**^**b**^	44.3	44.8	0.085	6.1	4.9	<0.001
**Vigorous exercise (%)**^**c**^	16.2	13.3	<0.001	13.8	14.8	0.007
**High education level (%)**^**d**^	85.2	87.4	<0.001	71.8	54.7	<0.001
**Diabetes (%)**	1.5	5.4	<0.001	0.7	8.2	<0.001
**Hypertension (%)**	12.0	21.9	<0.001	4.4	20.2	<0.001
**Systolic BP (mmHg)**^**a**^	114.9 (11.9)	118.7 (12.7)	<0.001	105.9 (12.4)	114.8 (15.5)	<0.001
**Diastolic BP (mmHg)**^**a**^	74.4 (8.9)	77.4 (9.5)	<0.001	67.5 (8.7)	73.2 (10.3)	<0.001
**Glucose (mg/dl)**^**a**^	93.1 (11.8)	98.7 (18.4)	<0.001	90.1 (9.1)	100.1 (22.7)	<0.001
**Uric acid (mg/dl)**^**a**^	5.9 (1.1)	6.5 (1.2)	<0.001	4.1 (0.8)	4.7 (1.0)	<0.001
**Total cholesterol (mg/dl)**^**a**^	192.3 (32.4)	208.4 (34.8)	<0.001	183.9 (31.9)	204.6 (36.4)	<0.001
**LDL-C (mg/dl)**^**a**^	114.8 (28.0)	128.7 (30.3)	<0.001	103.1 (27.0)	124.8 (31.8)	<0.001
**HDL-C (mg/dl)**^**a**^	54.1 (11.4)	48.0 (9.3)	<0.001	62.5 (13.4)	53.5 (11.7)	<0.001
**Triglycerides (mg/dl)**^**e**^	102 (76–141)	155 (113–214)	<0.001	72 (56–97)	122 (88–171)	<0.001
**ALT (U/l)**^**e**^	14 (11–20)	16 (11–22)	<0.001	14 (11–19)	14 (11–18)	<0.001
**AST (U/l)**^**e**^	18 (15–21)	18 (15–22)	<0.001	18 (15–21)	18 (15–21)	<0.001
**GGT (U/l)**^**e**^	16 (11–23)	17 (12–28)	<0.001	15 (11–22)	15 (11–20)	<0.001
**HOMA-IR**^**e**^	1.47 (1.04–1.96)	2.13 (1.55–2.82)	<0.001	1.45 (0.97–1.97)	2.27 (1.60–3.10)	<0.001
**hsCRP (mg/l)**^**e**^	0.4 (0.2–0.8)	0.7 (0.4–1.4)	<0.001	0.3 (0.1–0.6)	0.9 (0.4–1.8)	<0.001

Data are presented as ^a^mean (standard deviation), ^e^medians (interquartile range), or percentage.

ALT, alanine aminotransferase; BMI, body mass index; BP, blood pressure; HDL-C, high-density lipoprotein-cholesterol; hsCRP, high sensitivity C-reactive protein; HOMA-IR, homeostasis model assessment of insulin resistance.

^b^ ≥ 10 g of ethanol per day; ^c^ ≥ 3 times per week; ^d^≥ College graduate

The association between NAFLD and the development of nephrolithiasis was examined by sex. During 1,054,887.6 person-year of follow-up, 16,442 developed nephrolithiasis (overall incidence rate, 1.6 per 100 person-years; 1.8 per 100 person-years in men and 1.3 per 100 person-years in women). The median follow-up period for participants was 6.6 years (interquartile range 3.0–9.4 years). The risk for the development of nephrolithiasis varied significantly by sex (*p* for interaction <0.001). For men, NAFLD at baseline was associated with a significantly higher risk of nephrolithiasis than for those without NAFLD, after adjusting for age (adjusted HR 1.32, 95% CI 1.25–1.37). This association was still significant after adjustment for center, year of screening exam, smoking status, alcohol intake, physical activity, education level, BMI, history of hypertension and diabetes, HOMA-IR, uric acid, and hsCRP (Model 2) (adjusted HR 1.17, 95% CI 1.06–1.30). On the other hand, NAFLD was not significantly related to the risk for nephrolithiasis in women (adjusted HR 0.97, 95% CI 0.81–1.16) (Table 2).

**Table 2:** Development of nephrolithiasis by non-alcoholic fatty liver disease (NAFLD).

Presence of NAFLD	Person-year	Incident case	Incidence density (per 100 person-years)	Aged-adjusted HR (95% CI)	Multivariate HR^a^ (95% CI)	
					Model 1	Model 2
**Men**						
**No NAFLD**	459,878.1	6,061	1.3	1.00 (reference)	1.00 (reference)	1.00 (reference)
**NAFLD**	595,009.5	4,462	1.8	1.32 (1.27–1.37)	1.16 (1.11–1.22)	1.17 (1.06–1.30)
**Women**						
**No NAFLD**	417,820.6	5,198	1.2	1.00 (reference)	1.00 (reference)	1.00 (reference)
**NAFLD**	42,057.6	721	1.7	1.10 (1.01–1.19)	1.07 (0.97–1.17)	0.97 (0.81–1.16)

^a^Estimated from parametric Cox models. Multivariable model 1 was adjusted for age, center, year of screening exam, smoking status, alcohol intake, physical activity, education level, BMI, and history of hypertension and diabetes: Model 2: model 1 plus adjustment for HOMA-IR, uric acid, and hsCRP

BMI, body mass index; CI, confidence intervals; HR, hazard ratios.

p value <0.001 for the interaction between sex and NAFLD for the development of nephrolithiasis.

The association between NAFLD and nephrolithiasis was more prominent in participants less than 50 years of age (adjusted HR 1.19, 95%CI 1.14–1.24) than in those older than 50 (adjusted HR 1.06, 95% CI 0.95–1.19) (p for interaction<0.001).

Except for age, the associations between NAFLD and nephrolithiasis were similar among the subgroups of study participants. There were no significant associations with alcohol intake (<10 vs. ≥10g/day), smoking status (never vs. current smokers), HOMA-IR (<2.5 vs. ≥2.5), BMI (<25 vs. ≥25kg/m^2^), and hsCRP (<1.0 vs. ≥1.0mg/l) (Table 3).

**Table 3:** Hazard ratios^a^ (95% CI) for the development of nephrolithiasis according to non-alcoholic fatty liver disease (NAFLD) in clinically relevant subgroups.

Subgroup	No NAFLD	NAFLD	*p*-value for interaction
**Age**			<0.001
<50 years (N = 192,626)	Reference	1.19 (1.14–1.24)	
≥50 years (N = 15,952)	reference	1.06 (0.95–1.19)	
**Alcohol intake**			0.130
<10 g/day (N = 141,488)	reference	1.13 (1.07–1.19)	
≥10 g/day (N = 53,504)	Reference	1.18 (1.11–1.26)	
**Smoking**			0.223
Never (N = 151,496)	reference	1.14 (1.08–1.19)	
Current smoker (N = 49,209)	reference	1.17 (1.09–1.25)	
**HOMA-IR**			0.523
<2.5 (N = 170,774)	reference	1.16 (1.10–1.21)	
≥2.5 (N = 32,985)	reference	1.11 (1.02–1.20)	
**BMI**			0.684
<25 kg/m^2^ (N = 154,843)	reference	1.19 (1.13–1.25)	
≥25 kg/m^2^ (N = 53,718)	reference	1.14 (1.08–1.21)	
**hsCRP**			0.447
<1.0 mg/l (N = 157,588)	reference	1.14 (1.09–1.20)	
≥1.0 mg/l (N = 49,444)	reference	1.17 (1.09–1.25)	

^a^Estimated from parametric Cox models adjusted for age, sex, center, year of screening exam, smoking status, alcohol intake, physical activity, education level, BMI, and history of hypertension and diabetes

## Discussion

In this large cohort study of young and middle-aged Korean adults, NAFLD was significantly associated with the development of nephrolithiasis in men, whereas there was no significant association in women. For men, the modest association between NAFLD and incidence of nephrolithiasis persisted even after adjusting for possible confounders and metabolic factors, suggesting that NAFLD is an independent risk factor for nephrolithiasis in men. To the best of our knowledge, this is the first cohort study to demonstrate that NAFLD is associated with an increased risk of nephrolithiasis.

A study at an Iranian medical center reported that patients with NAFLD showed a higher prevalence of nephrolithiasis [13]. However, this study used a cross-sectional design and did not adjust for possible confounders such comorbidities, lifestyle factors, and anthropometric measures, limiting the temporal and independent association between NAFLD and the development of nephrolithiasis.

The mechanisms whereby NAFLD contributed to nephrolithiasis remain incompletely elucidated. NAFLD and nephrolithiasis share several risk factors such as obesity, hypertension, diabetes and MetS [10]. Insulin resistance, a key factor in the pathogenesis of NAFLD, contributes to the formation of kidney stones by affecting urinary pH [25]. Insulin receptors are expressed in the renal tubular epithelium and insulin participates in ammoniagenesis of the renal tubule [26]. Insulin resistance, a status of impaired insulin function, leads to decreased ammoniagenesis in the renal tubule [26, 27], resulting in acidic urine which may promote uric acid stones. In our study, the association between NAFLD and incident nephrolithiasis was evident even after adjustment for BMI, hypertension, diabetes, and HOMA-IR, and the association persisted among non-obese participants or among those with low hsCRP or with low HOMA-IR, suggesting that other mechanisms may influence the association between NAFLD and nephrolithiasis.

Kidney stone development can be attributed to reactive oxygen species (ROS) and inflammation [28]. Previous studies have suggested that stone formation can begin inside oxidatively damaged cells [29], leading to cell death and the formation of membrane-bound vesicles which induce crystal nucleation [30]. ROS initiate a signaling pathway that produces macromolecules to activate or inhibit crystal nucleation, growth, and aggregation [28]. Moreover, inflammatory markers and pro-inflammatory cytokines were found to be elevated in patients with nephrolithiasis [10, 31]. In addition, lipotoxicity may contribute to renal cell damage, impaired renal cell function, and decreased ammoniagenesis [32]. Increased levels of pro-inflammatory molecules and lipotoxicity are also features of NAFLD [33].

The association between NAFLD and nephrolithiasis was observed in men but not in women. These results are consistent with the previous study of Lonardo et al., which reported that risk factors for NAFLD vary according to sex [27]. The high prevalence of nephrolithiasis and NAFLD in men could partly explain this difference [5, 34]. There may also be other factors contributing to the occurrence of nephrolithiasis, such as estrogen status [35], which could attenuate the influence of NAFLD on formation of renal stones. Estrogen may protect against kidney stone formation [36]. On the other hand, decreasing estrogen levels are associated with worsened metabolic status in postmenopausal women, leading to the formation of nephrolithiasis [37]. In addition, menopausal status can accelerate bone turnover, causing increased urine calcium excretion and decreased citrate excretion [38]. Due to the small number of postmenopausal women in our study, we were not able to perform stratified analysis by menopausal status. Further research is needed to examine the association between nephrolithiasis and NAFLD in women.

Additionally, the association between NAFLD and nephrolithiasis was more evident in the NAFLD group younger than 50 years of age. It is possible that the age-related increase in comorbidities may confound the association. Another possible explanation for the differences among the subgroups may by chance.

There are some limitations of this study. First, we did not adjust for diet, which is an important risk factor of nephrolithiasis. Second, clinical data related to symptoms of nephrolithiasis were not available. However, abdominal US was performed routinely on all participants and nephrolithiasis was determined based on US findings. In this way we avoided recall bias and diagnosed asymptomatic nephrolithiasis. Third, information on the chemical composition of renal stones and specific renal stone type was not available for analysis. Fourth, we used abdominal US to detect nephrolithiasis even though unenhanced computed tomography (CT) has been reported to be more sensitive (>95%) and specific (>96%) for diagnosis of nephrolithiasis [39]. However, a study comparing US with CT reported that US has lower sensitivity and specificity (70.0% and 94.4%, respectively) than CT for detection of renal stones; thus, this could have led to misclassification of some participants with renal stones [40]. Also, we used US for the diagnosis of fatty liver, while liver biopsy is regarded as the reference standard. A meta-analysis revealed that the overall sensitivity and specificity of US for the detection of moderate-to-severe fatty liver compared to histology was 84.8% and 93.6%, respectively [23]. Although US assessment has an acceptable degree of diagnostic accuracy for steatosis, it cannot detect fatty infiltration below a threshold of 10% [41]. This type of error may result in underestimation of the true association between NAFLD and nephrolithiasis. Recently, semiquantitative US indices have been considered a reliable screening tool for metabolic derangements and might be helpful in understanding the pathogenesis of the NAFLD-nephrolithiasis association in future research, though these were not available in our study [42]. Further studies are needed using more sensitive and specific techniques to detect renal stones and NAFLD. Lastly, our study was conducted in asymptomatic young to middle-aged, apparently healthy Korean adults. Thus, our findings may not be applicable when generalized to other populations.

In conclusion, in a large cohort of Korean adults, NAFLD was associated with an increased risk for development of nephrolithiasis in men but not in women. This association persisted after controlling for possible confounders and other metabolic parameters, suggesting an independent role of NAFLD in the pathogenesis of nephrolithiasis.
